# Supplementary material for: The impact of long-term non-pharmaceutical interventions on COVID-19 epidemic dynamics and control
Source: medRxiv. 2020 May 6:2020.05.03.20089078. Preprint. [Version 1] doi: 10.1101/2020.05.03.20089078 (PMC7276010; doi:10.1101/2020.05.03.20089078)
Supplement: 1 [file NIHPP2020.05.03.20089078-supplement-1.pdf]

medRxiv preprint doi: https://doi.org/10.1101/2020.05.03.20089078.this version posted May 6, 2020. The copyright holder for this preprint (which was not certified by peer review) is the author/funder, who has granted medRxiv a license to display the preprint in perpetuity.
It is made available under a CC-BY 4.0 International license .

Supplemental Material for: The impact of long-term non-pharmaceutical interventions on COVID-19 epidemic dynamics and control

Marissa Childs[1]*, Morgan P. Kain[2,3]*, Devin Kirk[2,4], Mallory Harris[2], Lisa Couper[2], Nicole Nova[2], Isabel Delwel[2], Jacob Ritchie[5], Erin A. Mordecai[2]

*Denotes equal authorship. Corresponding authors: marissac@stanford.edu, morganpkain@gmail.com

[1]Emmett Interdisciplinary Program in Environment and Resources, Stanford University, Stanford, CA, 94305, USA

[2]Department of Biology, Stanford University, Stanford, CA, 94305, USA

[3]Natural Capital Project, Woods Institute for the Environment, Stanford University, Stanford, CA 94305, USA

[4]Department of Zoology, University of British Columbia, Vancouver, BC V6T 1Z4, Canada

[5]Department of Computer Science, Stanford University, Stanford, CA, 94305, USA

medRxiv preprint doi: https://doi.org/10.1101/2020.05.03.20089078.this version posted May 6, 2020. The copyright holder for this preprint (which was not certified by peer review) is the author/funder, who has granted medRxiv a license to display the preprint in perpetuity. It is made available under a CC-BY 4.0 International license .

Supplement: Equations and model implementation

We assume an underlying, unobserved process model of SARS-CoV-2 transmission described by Equation set S1 and shown in Figure S1. The compartments in the model are susceptible (S); exposed but not infectious (E); infectious and asymptomatic (I_A), presymptomatic (I_P), mildly symptomatic (I_M), and severely symptomatic (I_S); hospitalized cases that will recover (H_R) or die (H_D); and recovered (R). We use an Euler approximation of the continuous time process with a time step of 4 hours. Transitions between compartments are simulated as binomial ($\mathcal{B}$) or multinomial ($\mathcal{M}$) processes; Equation set S2 describes in detail the stochastic rates used to approximate the transition terms in Equation set S1. Parameters are defined in Tables 1 and 2. Finally, we assume that the observed deaths are a Poisson random variable with mean of total new deaths accumulated over the observation period (i.e. one day for this analysis).

$$
\begin{aligned}
\frac{dS}{dt} &= -dSE \\
\frac{dE}{dt} &= dSE - dEI_A - dEI_P \\
\frac{dI_a}{dt} &= dEI_A - dI_AR \\
\frac{dI_p}{dt} &= dEI_P - dI_PI_S - dI_PI_M \\
\frac{dI_m}{dt} &= dI_PI_M - dI_MR \\
\frac{dI_s}{dt} &= dI_PI_S - dI_SH_R - dI_SH_D \\
\frac{dH_R}{dt} &= dI_SH_R - dH_RR \\
\frac{dH_D}{dt} &= dI_SH_D - dH_DD \\
\frac{dR}{dt} &= dH_RR \\
\frac{dD}{dt} &= dH_DD
\end{aligned}
\tag{S1}
$$

medRxiv preprint doi: https://doi.org/10.1101/2020.05.03.20089078.this version posted May 6, 2020. The copyright holder for this preprint
(which was not certified by peer review) is the author/funder, who has granted medRxiv a license to display the preprint in perpetuity.
It is made available under a CC-BY 4.0 International license .

$$
\begin{aligned}
dSE &\sim \mathcal{B}\left(S, 1-\exp\left(-\beta_0 \frac{C_A I_A + C_P I_P + C_M I_M + C_S I_S}{N} dt\right)\right) \\
\begin{pmatrix} dEE \\ dEI_A \\ dEI_P \end{pmatrix} &\sim \mathcal{M}\left(E, \begin{pmatrix} \exp(-\gamma dt) \\ \alpha(1-\exp(-\gamma dt)) \\ (1-\alpha)(1-\exp(-\gamma dt)) \end{pmatrix}\right) \\
dI_A R &\sim \mathcal{B}(I_A, 1-\exp(-\lambda_A dt)) \\
\begin{pmatrix} dI_P I_P \\ dI_P I_M \\ dI_P I_S \end{pmatrix} &\sim \mathcal{M}\left(I_P, \begin{pmatrix} \exp(-\lambda_P dt) \\ \mu(1-\exp(-\lambda_P dt)) \\ (1-\mu)(1-\exp(-\lambda_P dt)) \end{pmatrix}\right) \\
dI_M R &\sim \mathcal{B}(I_M, 1-\exp(-\lambda_M dt)) \\
\begin{pmatrix} dI_S I_S \\ dI_S H_R \\ dI_S H_D \end{pmatrix} &= \mathcal{M}\left(I_S, \begin{pmatrix} \exp(-\lambda_S dt) \\ \delta(1-\exp(-\lambda_S dt)) \\ (1-\delta)(1-\exp(-\lambda_D dt)) \end{pmatrix}\right) \\
dH_R R &\sim \mathcal{B}(H_R, 1-\exp(-\rho_R dt)) \\
dH_D D &\sim \mathcal{B}(H_D, 1-\exp(-\rho_D dt))
\end{aligned}
\tag{S2}
$$

medRxiv preprint doi: https://doi.org/10.1101/2020.05.03.20089078.this version posted May 6, 2020. The copyright holder for this preprint
(which was not certified by peer review) is the author/funder, who has granted medRxiv a license to display the preprint in perpetuity.
It is made available under a CC-BY 4.0 International license .

Supplement: Figures

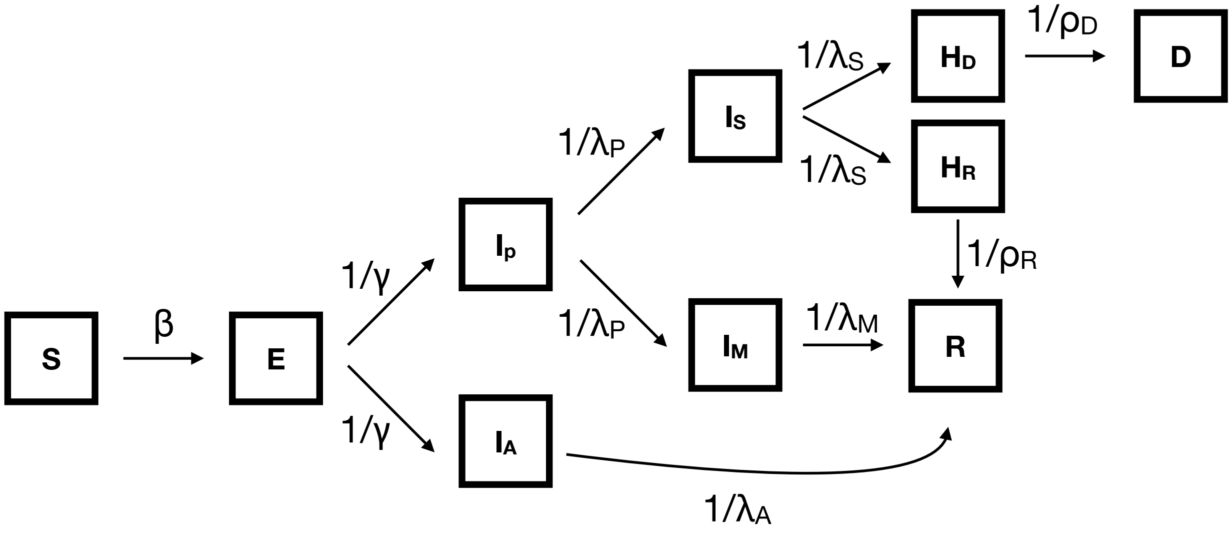

State variables

S = Susceptible
E = Exposed
I_P = Pre-symptomatic
I_A = Asymptomatic
I_S = Symptomatic, severe case
I_M = Symptomatic, mild case
H_D = Hospitalized, eventual death
H_R = Hospitalized, eventual recovery
R = Recovered
D = Dead

Transition Rates

β = Transmission rate
γ = Preinfectious period
λ_A = Asymptomatic infectious period
λ_P = Presymptomatic infectious period
λ_S = Severe infectious period until hospitalization
λ_M = Mild infectious period
ρ_D = Hospitalization period until death
ρ_R = Hospitalizations period until recovery

Figure S1: Epidemiological model box diagram

medRxiv preprint doi: https://doi.org/10.1101/2020.05.03.20089078.this version posted May 6, 2020. The copyright holder for this preprint
(which was not certified by peer review) is the author/funder, who has granted medRxiv a license to display the preprint in perpetuity.
It is made available under a CC-BY 4.0 International license .

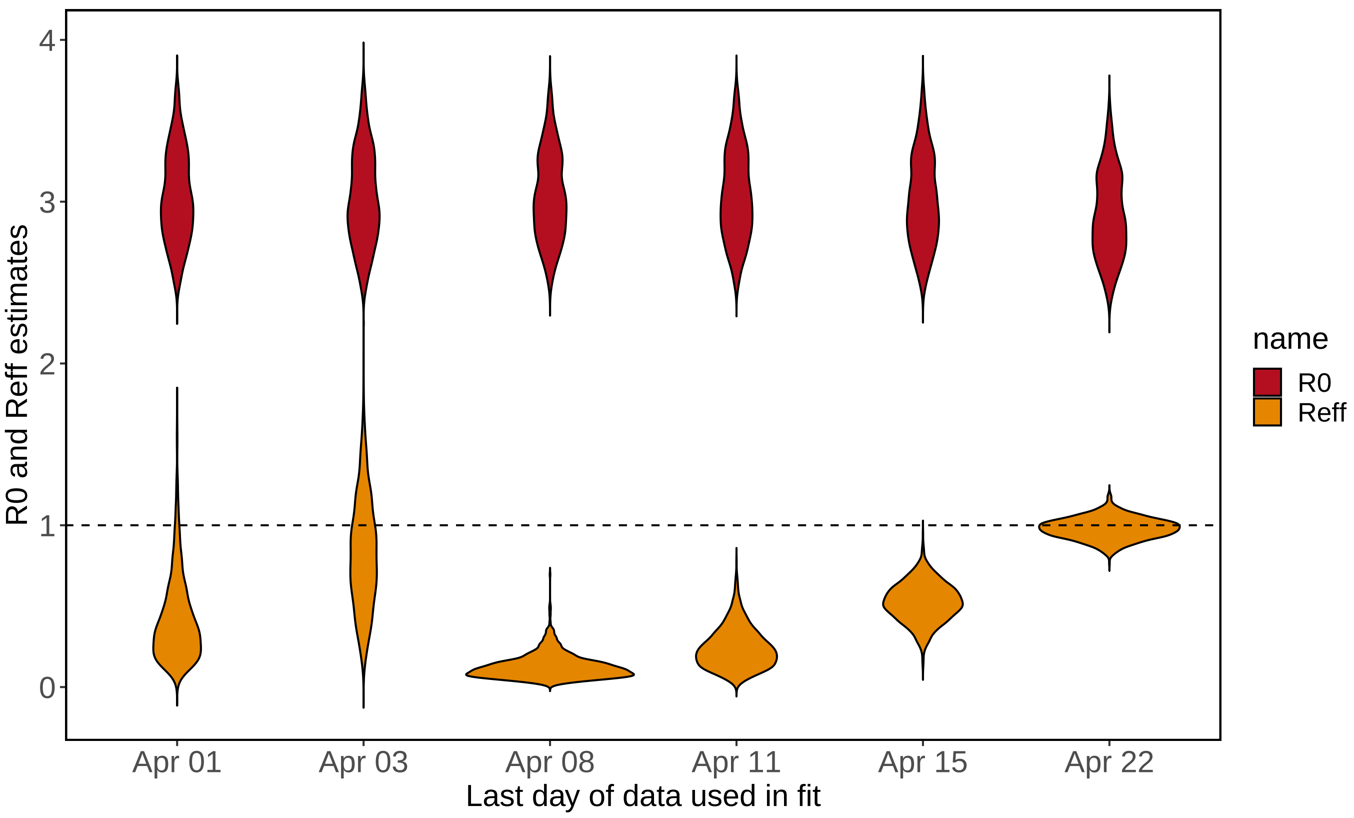

Figure S2: Estimated $\mathcal{R}_0$ and $\mathcal{R}_E$ from fits to truncated time series. A date corresponding to a pair of violin plots shows the most recent data for which data was used to fit the model. The low and confident $\mathcal{R}_E$ estimate on April 8 was due in part to 5 consecutive days, ending on April 8, with a total of 3 deaths.

medRxiv preprint doi: https://doi.org/10.1101/2020.05.03.20089078.this version posted May 6, 2020. The copyright holder for this preprint (which was not certified by peer review) is the author/funder, who has granted medRxiv a license to display the preprint in perpetuity.
It is made available under a CC-BY 4.0 International license .

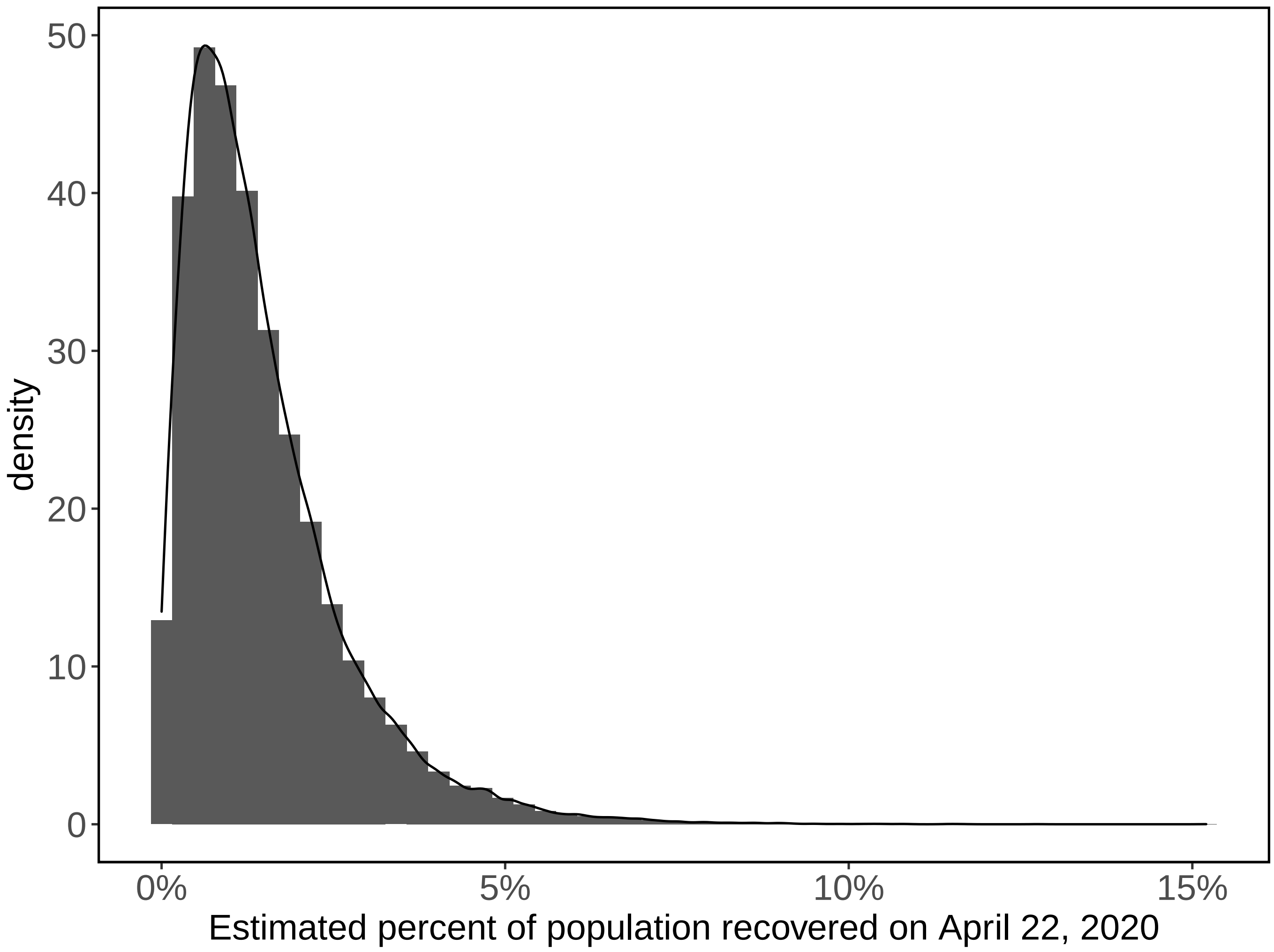

Figure S3: Distribution of estimated percent of Santa Clara County in the recovered class from 300 simulations of 200 parameter sets.

medRxiv preprint doi: https://doi.org/10.1101/2020.05.03.20089078.this version posted May 6, 2020. The copyright holder for this preprint
(which was not certified by peer review) is the author/funder, who has granted medRxiv a license to display the preprint in perpetuity.
It is made available under a CC-BY 4.0 International license .

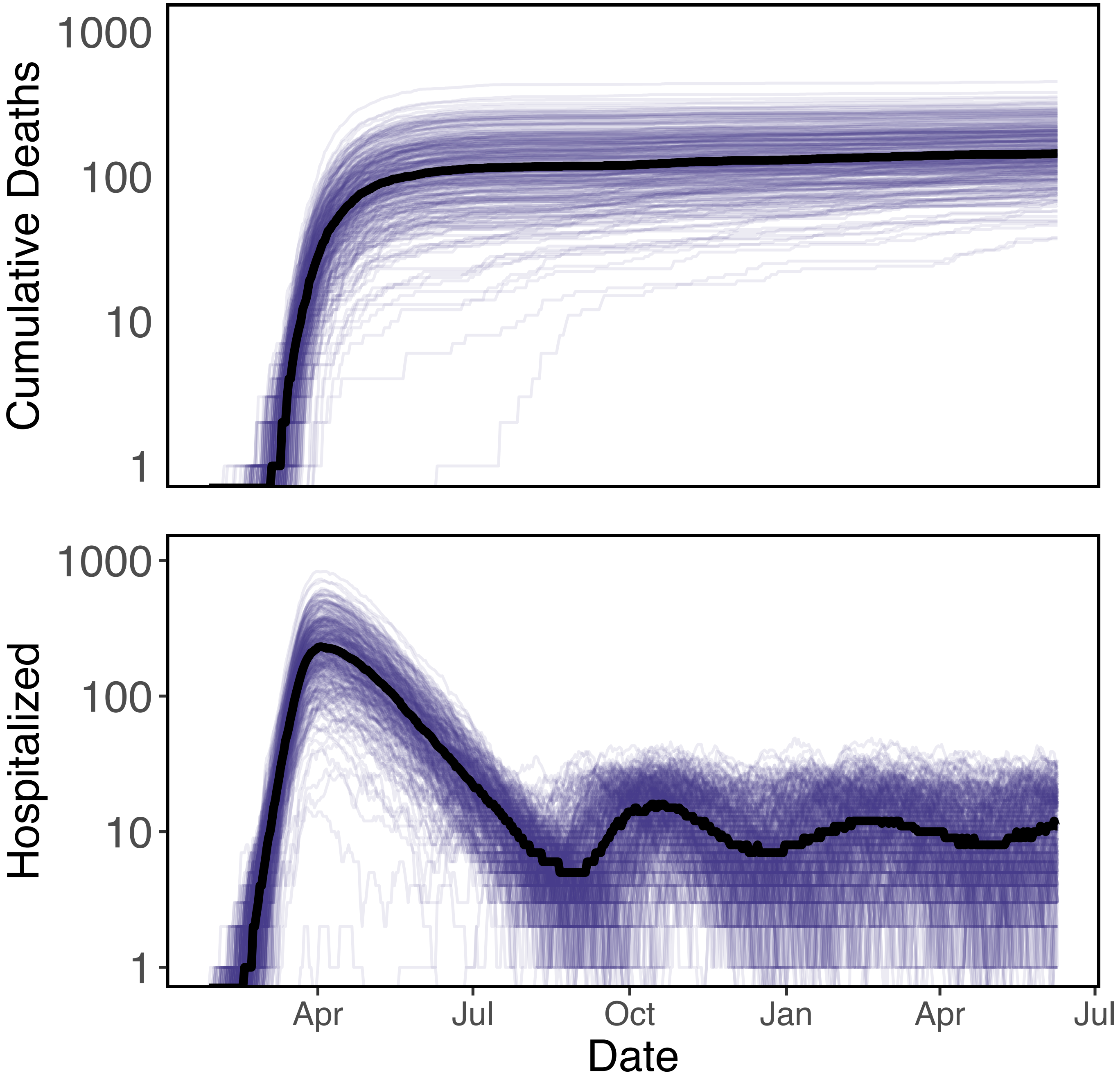

Figure S4: Adaptive triggering that alternates between a social distancing strength of 20% of background contacts and 50% of background contacts when the number of people hospitalized reaches 15 people or falls to 5 people, respectively. This strategy results in a moderately constant number hospitalized and leads to a slowly increasing cumulative death toll over time.
